# Delayed Revision Closed Reduction and Percutaneous Pinning of a Pediatric Supracondylar Humerus Fracture: A Case Report

**DOI:** 10.7759/cureus.70553

**Published:** 2024-09-30

**Authors:** Mohammed J Alanazi, Abdulaziz S AlTurki

**Affiliations:** 1 Division of Orthopedics, Department of Surgery, King Abdullah Bin Abdulaziz University Hospital, Princess Nourah Bint Abdulrahman University, Riyadh, SAU

**Keywords:** supracondylar fracture of the humerus (scfh), pediatric type iii supracondylar fractures, closed reduction and percutaneus pinning, revision, delayed presentation, supracondylar humeral fracture

## Abstract

Supracondylar humerus fractures (SCH) are the most prevalent elbow fractures in the pediatric age group. Delay in treatment poses challenges and an elevated risk of complications. We describe a case of revision for postoperative malalignment with closed reduction percutaneous pinning with good clinical outcomes. Malunion complications in SCH can be minimized with early intervention in cases of postoperative displacement. Careful use of technical skills can help with closed reduction in cases with delayed presentation.

## Introduction

In children, the most common fracture around the elbow is the supracondylar humerus fracture (SCH). Usually, these fractures result from falls from height [1]. In displaced patterns (Gartland II and III), treatment usually is with closed reduction and percutaneous pinning (CRPP) [2].

Delayed presentation (defined as a presentation after more than two days) poses treatment challenges. The elbow is severely swollen, and closed reduction is more difficult to perform. Complications in SCH are higher in cases of delayed presentation [3].

In operatively treated SCH fractures, the risk of early postoperative malalignment is around 5%. The most common malunion deformity is cubitus varus, which occurs in 10-30% of cases and usually results from medial comminution or posteromedial tilt of the distal fragment [4-7].

## Case presentation

The patient was a six-year-old boy, who was medically free. He sustained a fall and injured his left elbow. His father did manual traction, applied a home splint, and brought him to the ER. He was found to have a Gartland III SCH fracture with an inability to extend his fingers. His pulses were intact. He was treated with immediate closed reduction and Kirschner wire (K-wire) fixation and discharged home with persistent postoperative radial nerve palsy.

**Figure 1:**
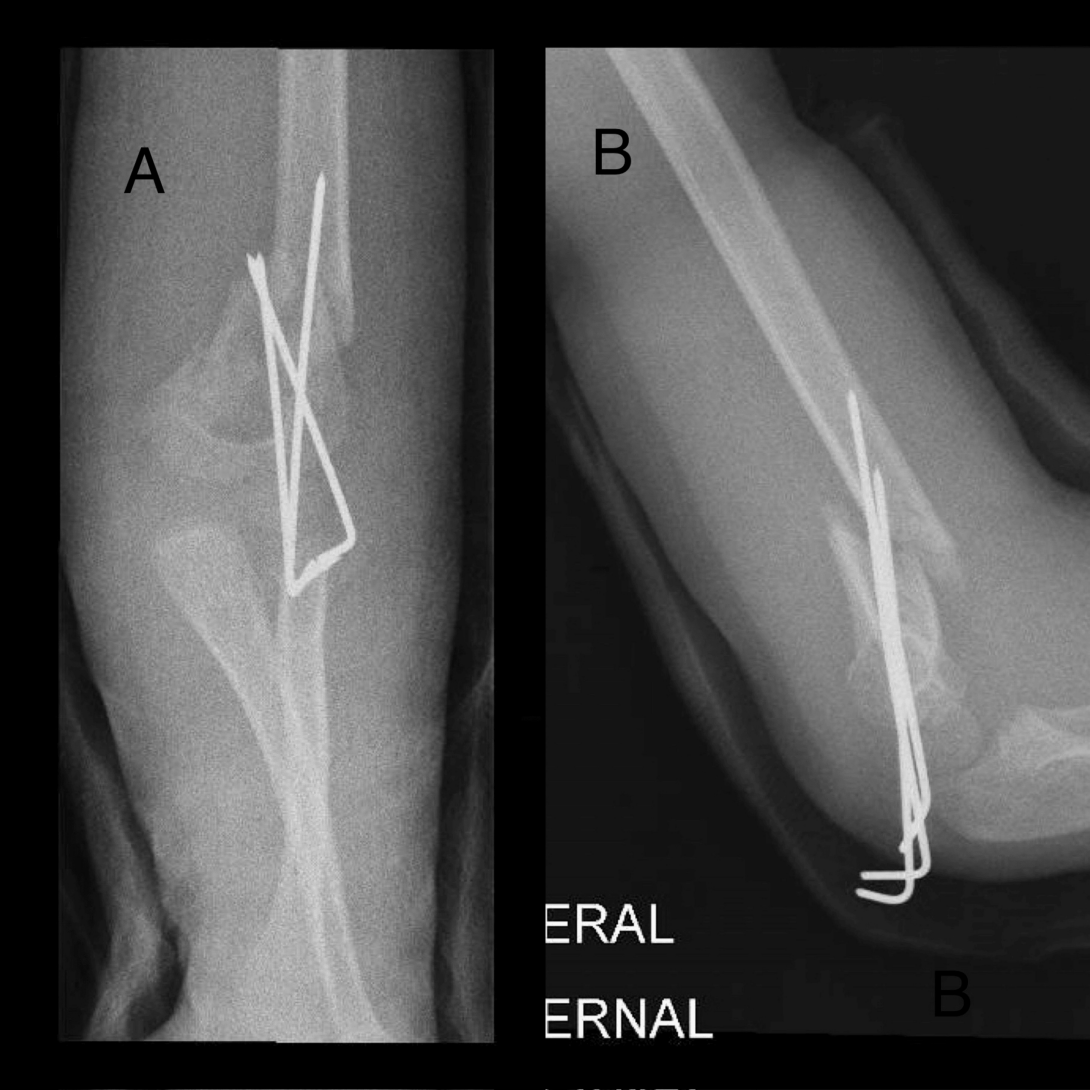
X-ray upon referral to our clinic (day 5 post initial CRPP) (A) anteroposterior view; (B) lateral view CRPP: closed reduction percutaneous pinning

**Figure 2:**
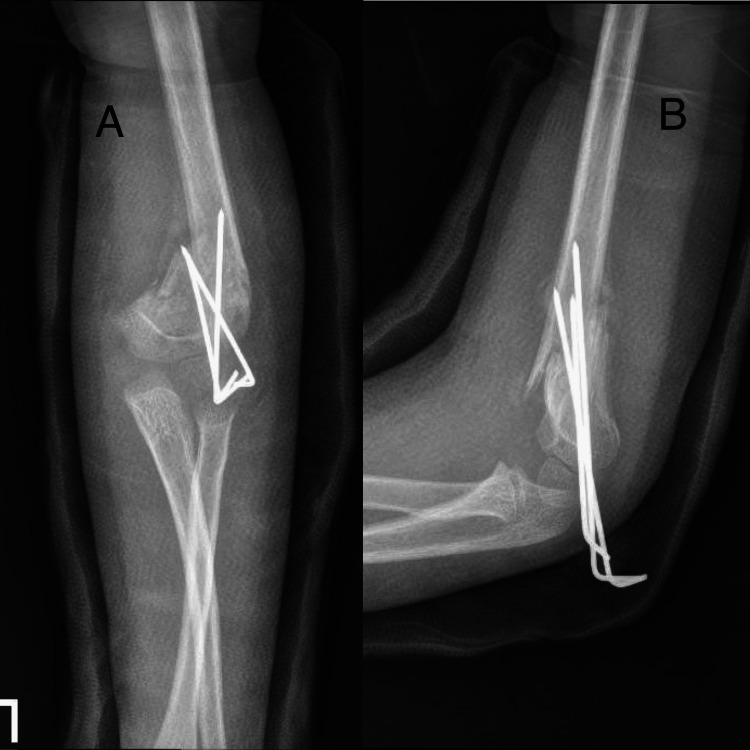
X-ray immediately before revision surgery (day 15 post initial CRPP) (A) anteroposterior view; (B) lateral view CRPP: closed reduction percutaneous pinning

On postoperative day 5, he was seen in the clinic, and displacement of the fracture was noted. At that time he was referred to us (Figure 1). After family counselling the decision was made to admit him immediately for revision. However, his admission was delayed because the patient tested positive for COVID-19. On postoperative day 15, the patient became COVID-19 negative and he was admitted and prepared for revision surgery (Figure 2).

He was reexamined at that time again and found to have an inability to extend or abduct his fingers. His pulses were strongly palpable. He consented to closed versus open reduction and K-wire fixation.

**Figure 3:**
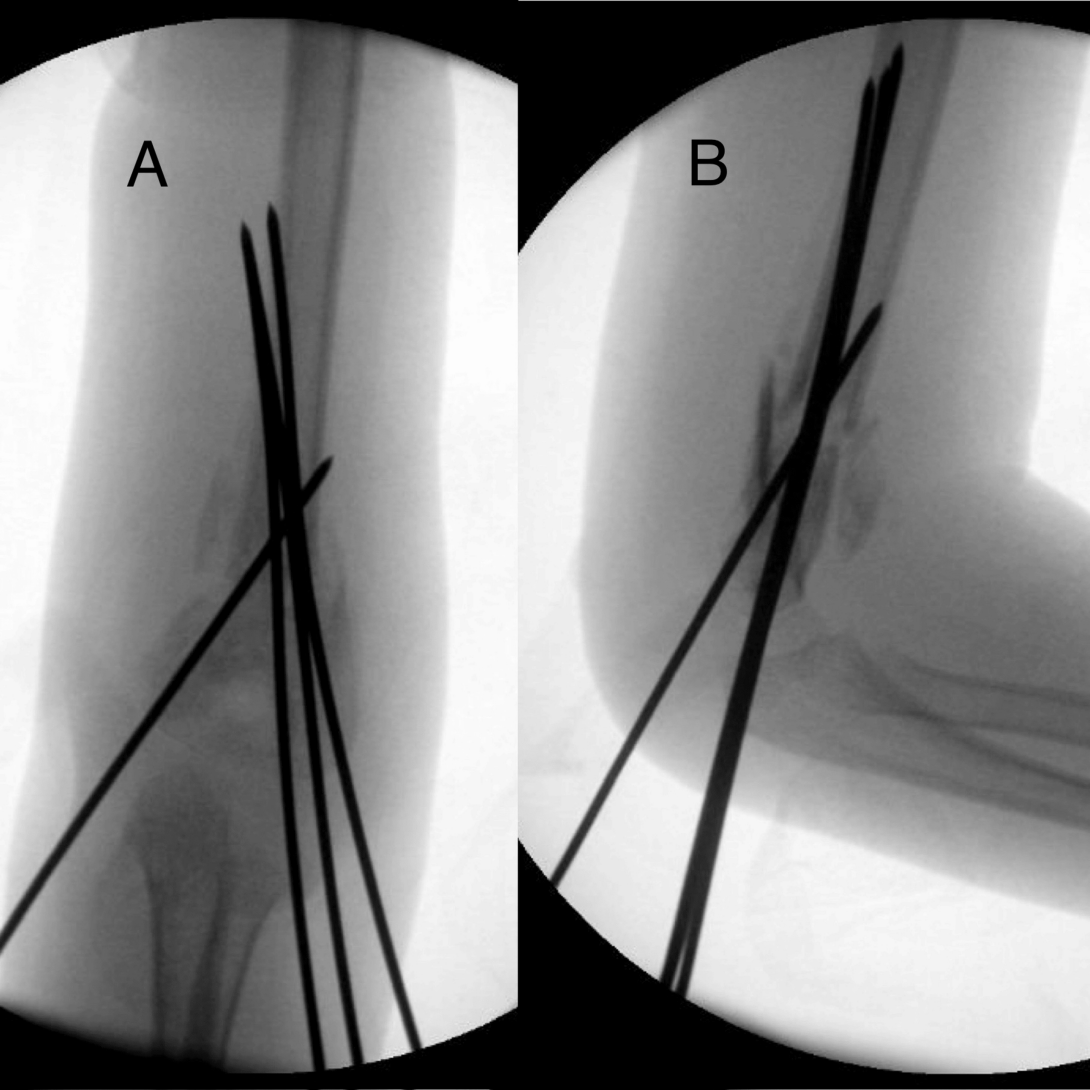
Intraoperative images of the revision CRPP (A) anteroposterior view; (B) lateral view CRPP: closed reduction percutaneous pinning

Closed reduction was attempted with gentle and sustained traction and counter traction but the fracture was “sticky”. A percutaneous K-wire was inserted through the triceps into the fracture site to break the callus. After osteoclasis was performed, the fracture became more mobile. Another K-wire was inserted from the lateral side as a joystick. Further manipulation with traction, pronation/supination/flexion was done. The fracture alignment improved. A small medial opening was made, anterior to midline with blunt dissection down to bone to protect the ulnar nerve. We manipulated the distal articular fragment with a K-wire. The alignment was acceptable so we inserted three K-wires from the lateral side and a medial wire for added stability. Live flouro was done and the fracture was relatively stable. Final X-rays showed acceptable reduction and proper placement of the wires (Figure 3). Standard postoperative care was done and pulses were palpable at procedure end.

**Figure 4:**
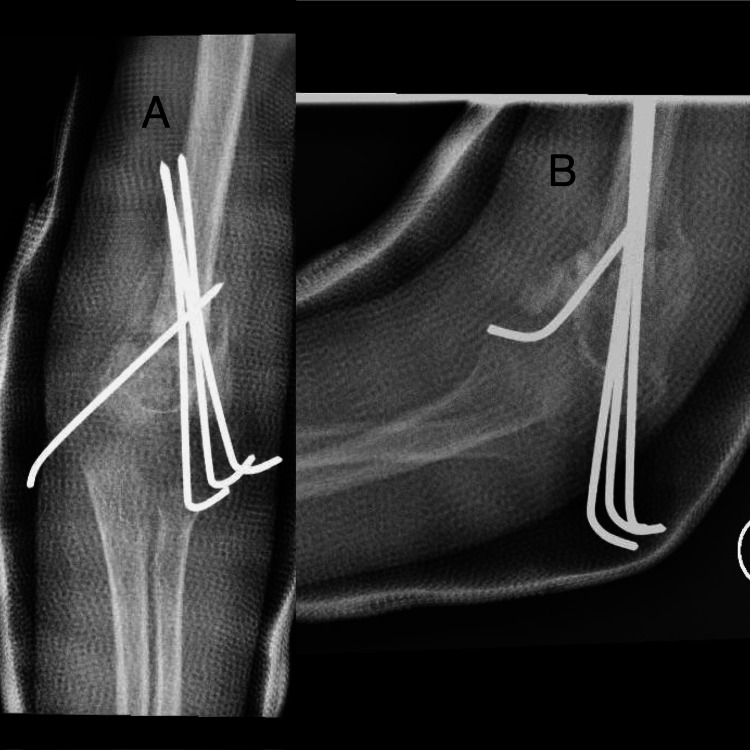
X-ray at the three-week follow-up (A) anteroposterior view; (B) lateral view

The patient was discharged the following day and his neurovascular exam remained the same as the preoperative. He was seen one week post surgery for a routine check-up. At the three-week follow-up, X-rays showed good healing (Figure 4). The wires were removed and range of motion (ROM) exercises were encouraged.

**Figure 5:**
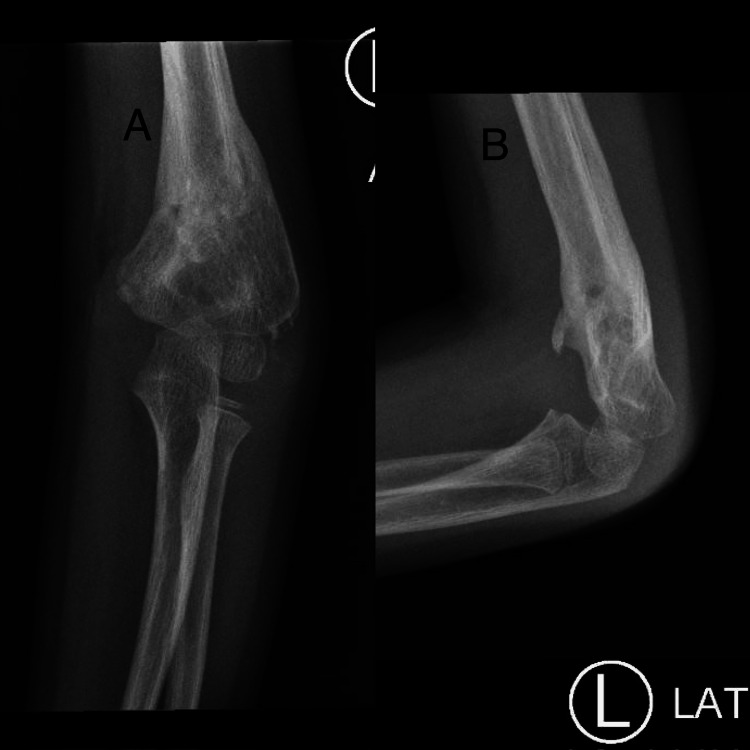
X-ray at the three-month follow-up (A) anteroposterior view; (B) lateral view

At the three-month follow-up, his nerve injuries were completely recovered and good fracture healing with mild cubitus varus was observed on X-rays. His ROM was normal (Figure 5).

**Figure 6:**
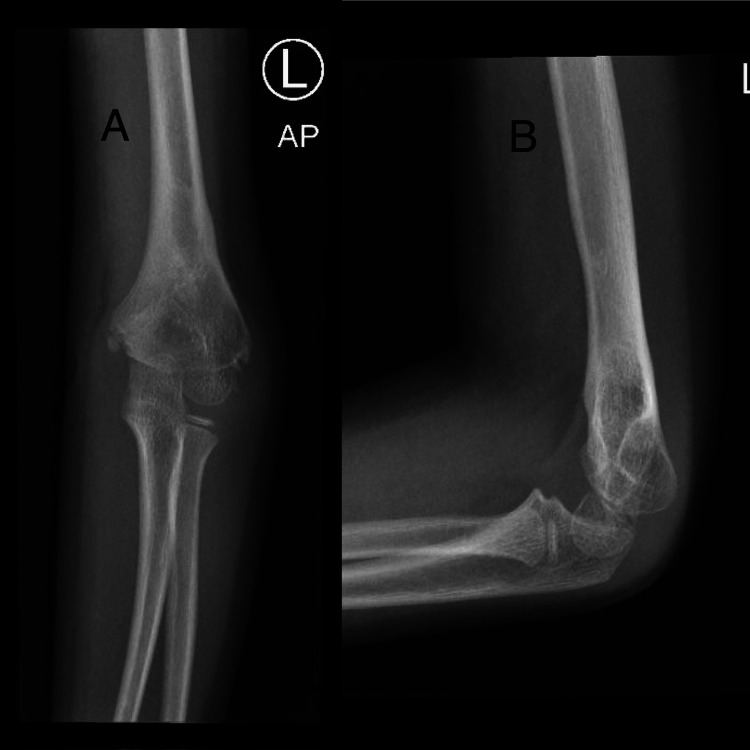
X-ray at the one-year follow-up (A) anteroposterior view; (B) lateral view

**Figure 7:**
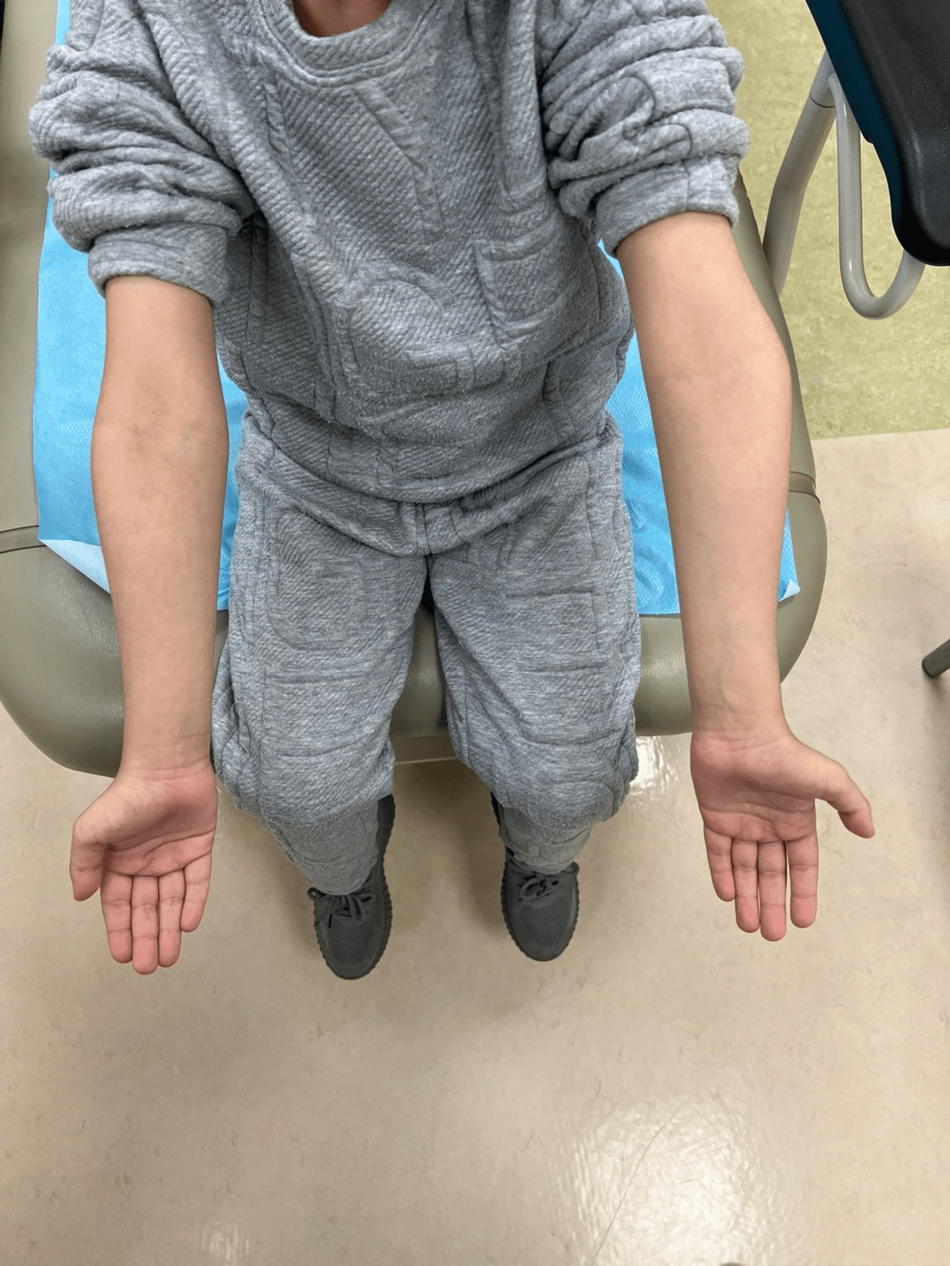
Clinical picture at the one-year follow-up

At the one-year postoperative follow-up, remodeling was noted on X-rays with stable mild cubitus varus (Figures 6, 7).

## Discussion

SCH fractures are common injuries. Displaced SCHs are generally treated with CRPP. Fracture displacement and loss of fixation in the early postoperative period are important to note to prevent late complications. A meta-analysis showed that deformity in SCH fractures occurred in 3.4% of the 849 patients treated with medial/lateral entry pins and in 5.9% of patients treated with lateral entry pins [3]. Wudbhav et al. described technical errors with pin placement that predispose to failure of fixation [8]. In their study, the incidence of fixation failure was 2.9%. Most of the failure occurred in Gartland III fractures that were treated with two lateral pins only. They recommended two lateral pins for Gartland II and three pins for Gartland III [8].

The concept of early revision is supported by literature as deformity correction later can have a high complication rate. In a study by Or et al. published in 2015, 396 patients with displaced SCH were treated surgically [5]. The incidence of early postoperative malalignment was 5%. They revised fixation for 21 patients within three weeks of the initial surgery due to malalignment. Their threshold to revise was malalignment of more than 10 degrees in the coronal or sagittal planes. They reported good outcomes of this early revision protocol with patients being pain-free, restoration of functional range of motion, and no significant cubitus varus at the final follow-up [5].

Liu et al. described a treatment plan for managed SCH fractures with delayed presentation by more than 14 days [1]. A total of 11 patients in their study were treated with minimally invasive reduction and external fixator. The external fixation was removed at the six-week follow-up. All patients had good to excellent outcomes on the Flynn criteria.

In our case, the postoperative displacement was significant and necessitated early intervention to minimize malunion deformity. The COVID-19 pandemic delayed our revision even further (16 days). However, with patience and percutaneous callus breakage, we managed to treat the patient with closed reduction. In revision cases and due to holes from the first surgery, the addition of more K-wires is necessary for fracture stability. The likelihood of needing open reduction is more in revision and delayed cases [9-12].

## Conclusions

SCH fractures are common injuries in children. In displaced fractures, the usual treatment is CRPP. Early postoperative malalignment can be managed with revision surgery or left to heal then managed later with corrective osteotomy. We present a case of delayed revision surgery with a good clinical outcome. We believe that even with delayed presentation, closed reduction remains the first option. Technical tricks and additional steps are usually needed to achieve satisfactory reduction with closed means.
